# Modelling time-varying volatility using GARCH models: evidence from the Indian stock market

**DOI:** 10.12688/f1000research.124998.1

**Published:** 2022-09-27

**Authors:** Farman Ali, Pradeep Suri, Tarunpreet Kaur, Deepa Bisht

**Affiliations:** 1 Department of Management, Uttaranchal University, Dehradun, Uttarakhand, 248001, India

**Keywords:** GARCH Model, Stock market, Volatility, NSE Return, Financial Crisis

## Abstract

**Background:** In this study, we examined the volatility of the Indian stock market from 2008 to 2021. Owing to the financial crisis, volatility forecasting of the Indian stock market has become crucial for economic and financial analysts. An empirical study of the returns of the NSE indices revealed an autoregressive conditional heteroskedastic trend in the Indian stock market.

**Methods:** Using GARCH 1, 1 (generalized autoregressive conditional heteroskedasticity) and FIGARCH (fractionally integrated GARCH), we examine investor behaviour and the persistence of long-term volatility.

**Results:** The empirical findings of the estimated models suggest that shocks persist for a long time in NSE returns. Furthermore, bad news has a greater impact on stock volatility than good news. The return on assets is stable but highly volatile, even though the Indian economy has experienced the global crash to some extent.

**Conclusions:** Models of volatility derived from the GARCH equation provide accurate forecasts and are useful for portfolio allocation, performance measurement, and option valuation.

## 1. Introduction

In recent years, both academics and financial analysts have shown an increasing interest in modelling and forecasting the volatility of financial time series, which is an increasingly fertile area of behavioural finance research. Since volatility affects many economic and financial applications, such as portfolio optimization, risk and returns analysis and asset pricing, it is a highly relevant concept. Asymmetry in the volatility process of unanticipated shocks is a prominent feature of financial market time series. Negative news often has a greater impact on the conditional variance of equity returns than positive news due to the leverage effect ( Black, 1976). Globalizing the interdependence and size of major financial markets, the transmission of financial market information to India has become a subject of interest. An economic spillover occurs when one event sets off another event in a similar way, impacting economies both within and outside the country ( Nandy & Chattopadhyay, 2019). In 2008, when Lehman Brothers collapsed in the US, a domino effect was created and hit economies worldwide, including India. Currently financial markets are closely interconnected and driven by trust ( Prasad & Reddy, 2009). In times of crisis, investors make errors of judgment as long as a group of investors make irrational decisions, leading to the worsening of the situation in the stock market. Developed and emerging markets have been a riveting field of research on behavioural finance owing to interlinked stock markets worldwide. Coronavirus disease (COVID-19) also causes a shock to the majority of countries because of the interconnected market ( Karkowska & Urjasz, 2021). The Indian stock market has thus far been resilient amidst the COVID-19 crosswind, despite the disrupting waves of the pandemic. According to Dhall & Singh (2020), the COVID-19 pandemic has induced herding behaviour at the industry level. The Indian economy witnessed a subsequent global crash to some extent. A study by Huang *et al.* (2020) concludes that foreign investors significantly increase crash risk in groups with low levels of real earnings management, while they have no significant effect on crash risk in groups with high levels of real earnings management. In India, it is very difficult to pinpoint the exact impact of the financial crisis, but it seems that the crisis has spilled over into some sectors. Recently, global regulatory lockdowns caused by COVID-19 have severely impacted both the real and financial sectors. In India, shock transmission has substantially increased, resulting in increased volatility. Fractionally integrated generalized autoregressive conditional heteroskedasticity (FIGARCH) has been applied to predict the persistence of the volatility of the Indian indices (NSE). A major goal of this study is to understand how one volatility index’s shocks can affect another’s volatility index.

This paper has the following structure. The literature review is in Section 2. The data and methodologies are presented in Section 3. In Section 4, we present our results. In Section 5, we summarize the paper.

## 2. Literature review

Recent studies have investigated the cointegration relationship of the stock market amid crises ( Aggarwal & Raja, 2019, Bouri *et al.*, 2017, Zhang & Wei, 2010). Some studies have explained the causal linkage while in several other studies authors have performed comprehensive analysis by applying the generalized autoregressive conditional heteroskedasticity (GARCH) 1,1 model developed by Bollerslev (1986). Recent developments in the field of behavioural finance have revealed the volatility clustering in the Indian stock market by analysing time series data. A GARCH model is believed to be extremely useful for modelling and forecasting asset return volatility over time ( Engle & Patton, 2001).

Maloney & Mulherin (2003) predicted investors’ irrational behaviour using a volatility forecasting model. Fehr & Tyran (2005) observed that a small group of irrational investors affects the aggregate outcome of the market during crashes; a small amount of individual irrationality may lead to large deviations from the aggregate predictions of rational models. Sadorsky (2006) found that for heating oil and natural gas, the TGARCH model (threshold GARCH model) fits well, whereas the GARCH model fits well for crude oil and unleaded gasoline. Alberg *et al*. (2008) analysed the dynamic nature of returns in terms of serial correlation, asymmetric volatility clustering, and leptokurtic innovation. Liu & Hung (2010) suggested that modelling the asymmetric component is more important than specifying the error distribution for enhancing volatility forecasts of financial returns when fat tails, leptokurtosis and leverage are present. Muthukumaran *et al.* (2011) argued that the Indian stock market was highly distressed by global financial crunches. Abdalla & Winker (2012) hypothesized that volatility and expected stock returns are positively correlated. Geels (2013) discussed the green growth discourse which resulted in a major green stimulus program, but this positive effect seems to be coming to an end. Lim & Sek (2013) analysed stock market volatility in Malaysia, finding that the GARCH model works well during a crisis while the TGARCH model works well in the post-crisis period. Asgharian *et al.* (2013) added the first principal component to the model, which outperformed all other specifications, demonstrating that the constructed principle component is a solid proxy for the economic cycle. Mahalingam & Selvam (2014), amid crises, hypothesized two different sample periods for Bombay stock exchange (BSE) index returns and found that more than 90% of the data were influenced by past values. Danso & Adomako (2014) identified the capital structure of firms in South Africa and found that Africa was not isolated from the impact of the 2007-2008 financial crises. Ding *et al.* (2014) demonstrated that the influence of the investor’s sentiment trend on stock returns build upon the direction of the investor’s sentiments change (optimistic or pessimistic). Labuschagne *et al.* (2015) argued that GARCH models produce more accurate results than risk-neutral historic distribution (RNHD) models for constant interest rates. Nowhere in the existing literature has it been mentioned that investors also act rationally during a crisis. Mamun *et al.* (2015) concluded that the investors have a greed of return, annoyance, and anger; again they are able to evaluate and take all of these behavioural emotions as well as certain key rational attributes in terms of their risk appetite. Bir *et al.* (2015) examined the volatility shocks in series, and found that volatility clusters are evident in empirical results. Balcilar *et al.* (2015) suggested that the causal relationships between oil prices are strong. During certain subperiods, but not all, both variables have predictive potential for each other. Molnár (2016) converted a GARCH (1,1) model to a range-GARCH (1,1) model. On 30 shares and six stock indices as well as simulated data, the range-GARCH model outperforms the standard GARCH model, both in terms of in-sample fit and out-of-sample forecasting. Akinsomi *et al.* (2018) observed the shifting of investors from anti-herding behaviour within the highly volatile market to herding behaviour within the low volatile market. An *et al.* (2018) found that firms located in countries with higher individualism have a higher stock price crash risk. Using GARCH, Wang *et al.* (2018) examined how the spillover effect varies over time. Singh (2019) studied the presence of the Monday effect in fear sentiments for all currency pairs, denoting high positive returns with substantial value, and the Friday effect displaying negative returns. GARCH and TGARCH ( Ben *et al.*, 2019) were used to analyse asymmetric volatility dynamics in major cryptocurrency markets. The conditional volatilities of equity indices show widespread evidence of asymmetry, structural changes spread to other markets with a big order of magnitude ( Harris *et al.*, 2019 *).* Hsu *et al.* (2020) suggested that negative stock-bond return correlations resulting from investor flight-to-safety become less prominent when stock market uncertainty indices are extremely high. Some studies also identified that the long-term volatility of the stock market depends upon many macroeconomic variables. ( Fang *et al.,* 2020) and Lyócsa & Molnár (2020) applied a nonlinear autoregressive model in which the autoregressive coefficient is determined by typical Google searches related to COVID-19 and observed volatility between November 2019 and May 2020. Previous studies ( Fang *et al.,* 2020) revealed that the autoregressive coefficient was negative throughout the whole event time; however, as market uncertainty and attention to the virus increased, the coefficient’s magnitude increased as well. Narasimha & Mushinada (2020) point out that investors are concerned about cognitive biases and therefore adapt to changing market dynamics. Vo (2020) investigated the strong positive correlation between foreign investors and crash risk due to asymmetric information in emerging markets. Among BRICS countries (Brazil, Russia, India, China, and South Africa) a diverse response to stock market volatility has been reported including negative and positive shocks ( Salisu & Gupta, 2021). Cui & Zhang (2020) suggested that negative information creates fear among investors, which leads to a larger stock price crash risk. Nikkinen & Peltomäki (2020) investigated the investors’ crash fears by analysing the published news on stock market shocks and developing complex associations between information and stock market returns. He *et al.* (2020) examined the ability of futures markets to price discoveries through margin trading in the stock market. Kumar & Misra (2020) found widespread evidence of long-term similarity between the NIFTY 50 index and the global market. Naik *et al.* (2020a) examined the robustness of GARCH for both heteroskedasticity and volatility clustering. Elyasiani *et al.* (2021) analysed market greed by incorporating the skewness index of investing, which captures investor excitement more than investor fear. Engle & Patton (2007) determined that the GARCH model allows long-term volatility predictions to be reliant on socioeconomic dynamics and provides estimates of volatility to be expected in a freshly launched market. Ghani *et al.* (2022) suggested that the economic policy uncertainty index has a predictive power to forecast. Even during COVID-19, numerous researchers applied the GARCH model; nonetheless, the hedging strategy was expensive, with oil providing maximum hedging effectiveness for Hong Kong ( Mensi *et al.*, 2022).

Previous studies have almost exclusively examined the integration of Asian economies with other developed countries such as the United States and Japan amidst crises. It was reported in the literature ( Salisu & Akanni, 2020) that during the crisis, the domino effects hit economies worldwide in the short-run and long-run. This is inconsistent with the arguments given by Aggarwal & Raja (2019), Rajwani & Mukherjee (2013) and Menon *et al.* (2009).

Although most of the current economic crisis has passed, further studies are still required to address developed and emerging Asian markets. To assess the persistence of volatility the FIGARCH model is applied to the Indian stock market. It is necessary to investigate whether the effect persists over time and, if so, for how long? Does the variance of the forecast error in one market change due to a shock in another market? Contributing to existing theory and strategic financial decision-making for investors, this study offers valuable insights. Since the economy is expected to grow rapidly and foreign investors are becoming increasingly interested in the country, it is imperative to understand how market volatility in India varies over time, persists, and is predictable. This may be useful in order to formulate hedging strategies and diversify international portfolios.

## 3. Methods

In our study, we used data from the NSE-NIFTY 50 (National Stock Exchange) indices. We collected data from two websites ^1^ for the long run; covering most of the recession from January 1, 2008 to December 2, 2021. The period of study is based on the daily return. We calculated the daily return by applying the [Return = log (The closing price of indices/Closing price of indices (-1))] equation to the closing price. We analysed the data using the EViews 12 software package.

### 3.1 Hypothesis

H0: Error variance has homoscedastic properties (no ARCH effect) as followed by Lim & Sek (2013), Donadelli *et al.* (2017), Naik *et al*. (2020b), Chowdhury *et al*. (2020) and Dai *et al.* (2021).

H1: Error variance is heteroscedastic (ARCH effects).

The autoregressive Conditional Heteroscedasticity (ARCH) ^2^ model of volatility explains that heteroscedasticity may be autocorrelated over time. Conditional informs that variance depends on errors made in the past; heteroscedasticity means unequal variance. This model was proposed by Noble Prize winners ( Engle *et al.*, 1987). Suppose that the variance is *y*_*t*_. The model is conditional for the variance *y*_*t*_ on *y*_*t*_-1, thus

$$\mathrm{Var}\;\left(y_{t}|y_{t-1}\right)=\sigma_{t}^{2}=\alpha_{0}+\alpha_{1}y_{t-1}^{2}$$ (1)

We have included the $\alpha_{0}$ ≥ zero (0) and $\alpha_{1}$ ≥ zero (0) to remove the (-) variance. In a series if the mean is equal to zero (this can be achieved by centring), then the model can be expressed as follows:

$$y_{t}=\sigma_{t}\;\epsilon_{t},$$ (2)

$$\begin{array}{c} \text{With}\;\sigma_{t}=\sqrt{\alpha_{0}+\alpha_{1}y_{t-1}^{2}},\\ \text{and}\;\epsilon_{t}\overset{\mathrm{iid}}{∼}\left(\mu =0,\sigma^{2}=1\right) \end{array}$$

In ARCH (1) model equation (2) $y_{t}^{2}\;\mathrm{has}\;\text{the}\;\mathrm{AR}\;\left(1\right)$ model

$$y_{t}^{2}=\alpha_{0}+\alpha_{1}y_{t-1}^{2}+\text{error}$$ (3)

(a) A causal model can only be transformed into a legitimate infinite order MA only when $\alpha_{1}^{2}<\frac{1}{3}$

(b)
$y_{t}\text{ is white noise when }0\leq \alpha 1\leq 1.$

ARCH ( *m*) process variance at a time is dependent upon observations at previous *m* times.

$$\;\mathrm{Var}\left(y_{t}|y_{t-1},\ldots ,y_{t-m}\right)=\sigma_{t}^{2}=\alpha_{0}+\alpha_{1}y_{t-1}^{2}+\cdots +\alpha_{m}y_{t-m}^{2}.$$ (4)

In theory, *y* *_t_* series squared will be AR ( *m*) with certain constraints applied to coefficients. GARCH models use past squared observations and past variances to calculate variances over time. The model (GARCH 1,1) can be defined as

$$\sigma_{t}^{2}=\alpha_{0}+\alpha_{1}y_{t-1}^{2}+\beta_{1}\sigma_{t-1}^{2}$$ (5)

Covariance stationery observed with the GARCH (1,1) model, this is *α*_1_ + *β*_1_ < 1.

The leverage effect predicts that an asset’s price will become more volatile when its price decreases. In response to ‘bad news’, volatility tends to rise, and volatility tends to fall in response to ‘good news’. This is due to financial and operating leverage ( Nelson, 1991). A simple variance specification of exponential GARCH is given by:

$$\log\sigma_{t}^{2}=\omega +\beta \log\sigma_{t-1}^{2}+a|\left|\frac{\varepsilon_{t-1}}{\sigma_{t-1}}\right|+\gamma \frac{\varepsilon_{t-1}}{\sigma_{t-1}}$$ (6)

The logarithmic form of the conditional variance implies that the leverage effect is exponential and that forecasts of variance are not negative. This hypothesis can be tested to determine whether there is a leverage effect. *γ* > 0 If *γ* ≠ 0, then the impact is asymmetric.

Furthermore, the TGARCH model was introduced by Zakoian (1994). The conditional variance of stock market index returns is based on the assumption that unexpected changes in index returns have different effects on the conditional variance of the index returns. Threshold GARCH is a combination of ARCH and GARCH models. It specifies the conditional variance as follows:

$$\sigma_{t}^{2}=\omega +\sum_{i=1}^{q}a_{i}\varepsilon_{t-i}^{2}+{\gamma \varepsilon }_{t-1}^{2}d_{t-1}+\sum_{j=1}^{p}\beta_{j}\sigma_{t-j}^{2}$$ (7)

where *d _t_* = 1 if *ε _t_* < 0 and *d _t_* = 0.

In this model, the good news ( *ε _t_* > 0) and bad news ( *ε _t_* < 0) have differential effects on the conditional variance. Good news has an impact *a*, while bad news has an impact *a* + *γ* it. If *γ* > 0 then the leverage effect exists and bad news increases volatility, while if *γ* ≠ 0 the news impact is asymmetric.

The FIGARCH model modifies this specification by incorporating a fractional difference term ( Baillie & Morana, 2009). This variance can be expressed as:

$$\sigma_{t}^{2}=\omega +\left(1-\beta (L\right)-\emptyset \left(L\right)\pi \left(L\right){\in_{t}^{2}}_{-1}+\beta \left(L\right)\sigma_{t-1}^{2}$$ (8)

### 3.2 Descriptive statistics

**Figure 1.**
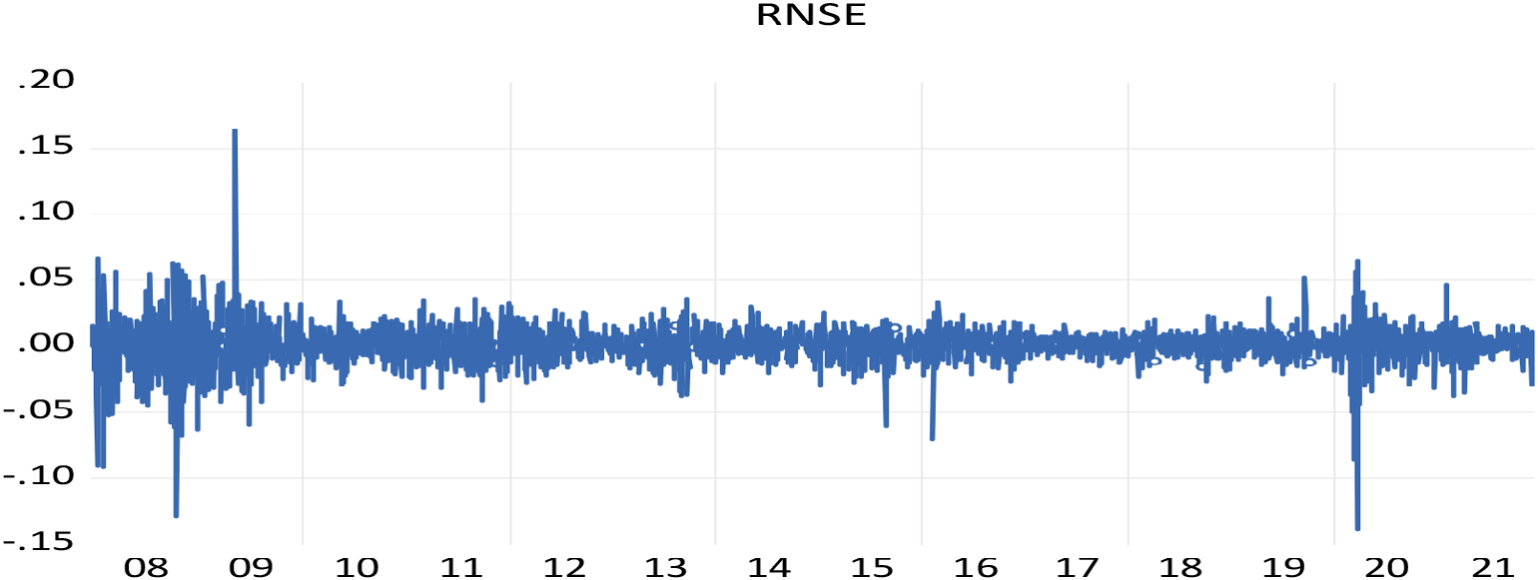
The volatility clustering of NSE return (source: author’s calculation). NSE: National Stock Exchange; RNSE: returns of NSE.

The NSE shows large shifts during times of crisis, followed by large shifts in the opposite direction, representing the wild and calm periods of volatility clustering. Figure 1 displays a clustering of volatility for NSE returns on a daily basis, as demonstrated by Mandelbrot & Taylor (1967) where “large changes tend to be followed by large changes, of either sign, and small changes tend to be followed by small changes.”

**Figure 2.**
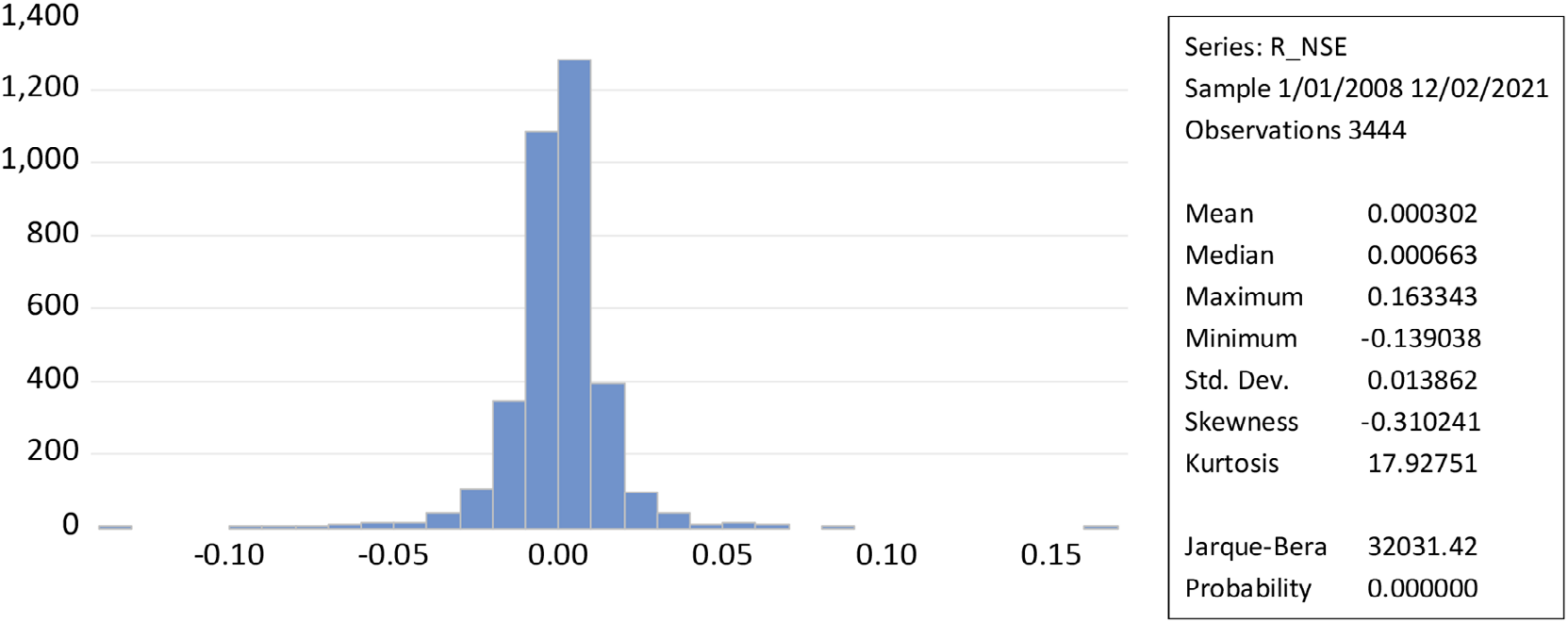
Time series and histogram plot of returns to NSE (source: author’s calculations). NSE: National Stock Exchange; RNSE: returns of NSE.

Figure 2 illustrates that leptokurtic statistical distributions with kurtosis larger than three result in a greater degree of stock market volatility. While the daily return of the NSE’s Jarque-Bera (32031.42) measures the market’s high volatility, the form of the curve, the magnitude of kurtosis, and the low probability value suggest that it may be possible to reject the null hypothesis.

In Table 1, the least square method is used to identify correlations between the dependent variable and independent variables by combining the mean and median, and the GARCH model is used to calculate the error distribution.

**Table 1.** The least square coefficients of NSE daily return (dependent variable: RNSE).

Variable	Coefficient	Std. error	t-Statistic	Prob.
C	0.000292	0.000236	1.234557	0.2171
RNSE (-1)	0.030297	0.017041	1.777853	0.0755

Source: author’s calculations. NSE: National Stock Exchange; RNSE: returns of NSE.

Table 2 shows that the statistics (73.209) and probability value (0.00) are statistically significant for the presence of ARCH effects. It is also estimated the value of α1=0.145, this indicates that the null hypothesis has been rejected. The heteroscedasticity test confirmed the existence of ARCH effects in the Indian stock market.

**Table 2.** Heteroskedasticity Test: ARCH. dependent variable; RESID^2 (method: least squares).

F-statistic	74.75665	Prob. F (1,3440)	0.0000
Obs*R-squared	73.20917	Prob. Chi-Square (1)	0.0000

Variable	Coefficient	Std. error	t-Statistic	Prob.
C	0.000164	1.37E-05	11.95115	0.0000
RESID^2(-1)	0.145839	0.016867	8.646193	0.0000

Source: author’s calculations. ARCH: autoregressive conditional heteroskedasticity.

Table 3 reveals the variance equation hence the GARCH (1,1) model is justified for the presence of time-varying conditional volatility of NSE returns.

**Table 3.** GARCH (1,1) statistics for the daily returns of NSE.

Dependent variable: RNSE				
Pre sample variance: backcast (parameter = 0.7)				
GARCH = C (3) + C (4) *RESID (-1) ^2 + C (5) *GARCH (-1)				
Variable	Coefficient	Std. error	z-Statistic	Prob.
C	0.000691	0.000154	4.473434	0.0000
RNSE (-1)	0.060154	0.018635	3.227959	0.0012
**Variance equation**				
C	1.75E-06	2.97E-07	5.898412	0.0000
RESID (-1) ^2	0.098858	0.006572	15.04136	0.0000
GARCH (-1)	0.893763	0.007090	126.0605	0.0000

Source: author’s calculations. GARCH: generalized autoregressive conditional heteroskedasticity; NSE: National Stock Exchange; RNSE: returns of NSE.

The mean equation from Table 3 can be derived as:

R_NSE=0.000691+0.060154 (9)

At present, the average return of the NSE is 0.000691 and its past value significantly forecasts the current series by 0.0601.

The coefficients were positive and statistically significant. We obtained the following variance equation for the NSE return:

$$\sigma_{t}^{2}=\alpha_{0}+\alpha_{1}y_{t-1}^{2}+\beta_{1}\sigma_{t-1}^{2}$$

R_NSE=0.00000175+0.893+0.098 (10)

For the long term with constant variances, the GARCH and ARCH parameters are statistically significant at the 1% level ( Table 3). The results of the GARCH model are as follows. The time-varying volatility of NSE daily returns includes a constant (0.00000175), past errors (0.893), and a component that depends upon past errors (0.098).

The GARCH (1,1) model and ARCH parameters indicate the persistence of volatility shocks. As a consequence, today’s shock is implied to remain in the forecast for years to come. In addition, we also examined the long-term dynamics of the Indian stock market using the FIGARCH. The lagged volatility and fitted variance are confirmed from the estimation output, and when comparing Table 3 and Table 4 GARCH and FIGARCH, we can see that the ARCH coefficient increases away from 1.00 whereas the GARCH coefficient decreases away from 1.00. This result highlights the long-term persistence of volatility shock in the Indian stock markets. Moreover, we analysed the impact of good and bad news on the volatility of the Indian stock market using the TGARCH and exponential GARCH (EGARCH) models. The multiplicative dummy variable ( Table 5) was added to the GARCH model to identify statistically significant differences when the shocks were negative.

**Table 4.** The outcome of FIGARCH for the daily returns of NSE.

Dependent variable: RNSE				
Method: ML ARCH - normal distribution (BFGS/Marquardt steps)				
GARCH = C (3) + C (4) *RESID (-1) ^2 + C (5) *GARCH (-1)				
Variable	Coefficient	Std. error	z-Statistic	Prob.
C	0.000702	0.000152	4.613667	0.0000
RNSE (-1)	0.063443	0.018065	3.511953	0.0004
**Variance equation**				
C (3)	3.44E-06	6.17E-07	5.566320	0.0000
RESID (-1) ^2	0.066285	0.027797	2.384627	0.0171
GARCH (-1)	0.649706	0.050215	12.93856	0.0000
D	0.636527	0.055110	11.55020	0.0000

Source: author’s calculations. FIGARCH: fractionally integrated generalized autoregressive conditional heteroskedasticity; GARCH: generalized autoregressive conditional heteroskedasticity; ML ARCH: Lagrange multiplier autoregressive conditional heteroskedasticity; NSE: National Stock Exchange; RNSE: returns of NSE.

**Table 5.** The results of TGARCH.

Dependent variable: RNSE				
Method: ML ARCH - normal distribution (Marquardt/EViews legacy)				
GARCH = C (3) + C (4) *RESID (-1) ^2 + C (5) *RESID (-1) ^2*(RESID (-1) <0) + C (6) *GARCH (-1)				
Variable	Coefficient	Std. error	z-Statistic	Prob.
C	0.000425	0.000152	2.806794	0.0050
RNSE (-1)	0.081077	0.015409	5.261564	0.0000
**Variance equation**				
C	3.77E-06	3.03E-07	12.44776	0.0000
RESID (-1) ^2	-0.001007	3.49E-12	-2.89E+08	0.0000
RESID (-1) ^2*(RESID (-1) <0)	0.193074	0.011060	17.45670	0.0000
GARCH (-1)	0.874378	0.005747	152.1319	0.0000

Source: author’s calculations. GARCH: generalized autoregressive conditional heteroskedasticity; ML ARCH: Lagrange multiplier autoregressive conditional heteroskedasticity; RNSE: returns of National Stock Exchange; TGARCH: threshold GARCH.

Nevertheless, the volatility behaviour of market index returns varies across market stages, and the Indian stock market has undergone various stages of development. We estimate the time varying volatility of positive shock; $\sigma_{t}^{2}$ = 0.00000377 + 0.874 + (-0.001).

Similarly, we estimate the time varying volatility of negative shock

$$\;\sigma_{t}^{2}=0.00000377+0.874+\left(-0.001+0.193\right)$$ (11)

The difference between good and bad news in the NSE index is 0.193 which is the coefficient of the asymmetric term. The coefficient of the asymmetric term is negative ( Table 6) and statistically significant at 1% level. In exponential terms this indicates that bad news has a greater effect on the volatility of NSE than good news.

**Table 6.** Estimating the results of EGARCH.

Dependent variable: RNSE				
LOG (GARCH) = C (3) + C (4) *ABS (RESID (-1)/@SQRT (GARCH (-1))) +				
C (5) *RESID (-1)/@SQRT (GARCH (-1)) + C (6) *LOG (GARCH (-1))				
Variable	Coefficient	Std. error	z-Statistic	Prob.
C	0.000306	0.000144	2.128865	0.0333
RNSE (-1)	0.075119	0.018037	4.164641	0.0000
C (3)	-0.276483	0.025319	-10.91984	0.0000
C (4)	0.163321	0.010552	15.47766	0.0000
C (5)	-0.105789	0.006731	-15.71784	0.0000
C (6)	0.983311	0.002144	458.5340	0.0000
R-squared	-0.001095	Mean dependent var		0.000301
Adjusted R-squared	-0.001386	S.D. dependent var		0.013864
S.E. of regression	0.013873	Akaike info criterion		-6.241322
Sum squared residual	0.662295	Schwarz criterion		-6.230615
Log likelihood	10750.44	Hannan-Quinn criter.		-6.237497
Durbin-Watson stat	2.088801			

EGARCH: exponential generalized autoregressive conditional heteroskedasticity; GARCH: generalized autoregressive conditional heteroskedasticity; RNSE: returns of National Stock Exchange; TGARCH: threshold GARCH.

As previously discussed, the basic GARCH model assumes that positive and negative shocks of the same absolute magnitude have the same impact on future conditional variances in the Indian stock markets. In contrast, previous studies ( Alberg *et al.*, 2008, Labuschagne *et al.,* 2015, Mathur *et al.*, 2016) have shown that the volatility of aggregate equity index returns can respond asymmetrically to past negative and positive returns, with poor returns resulting in higher volatility in the future. In economics, this is often referred to as the “leverage effect.”

## 4. Results

**Figure 3.**
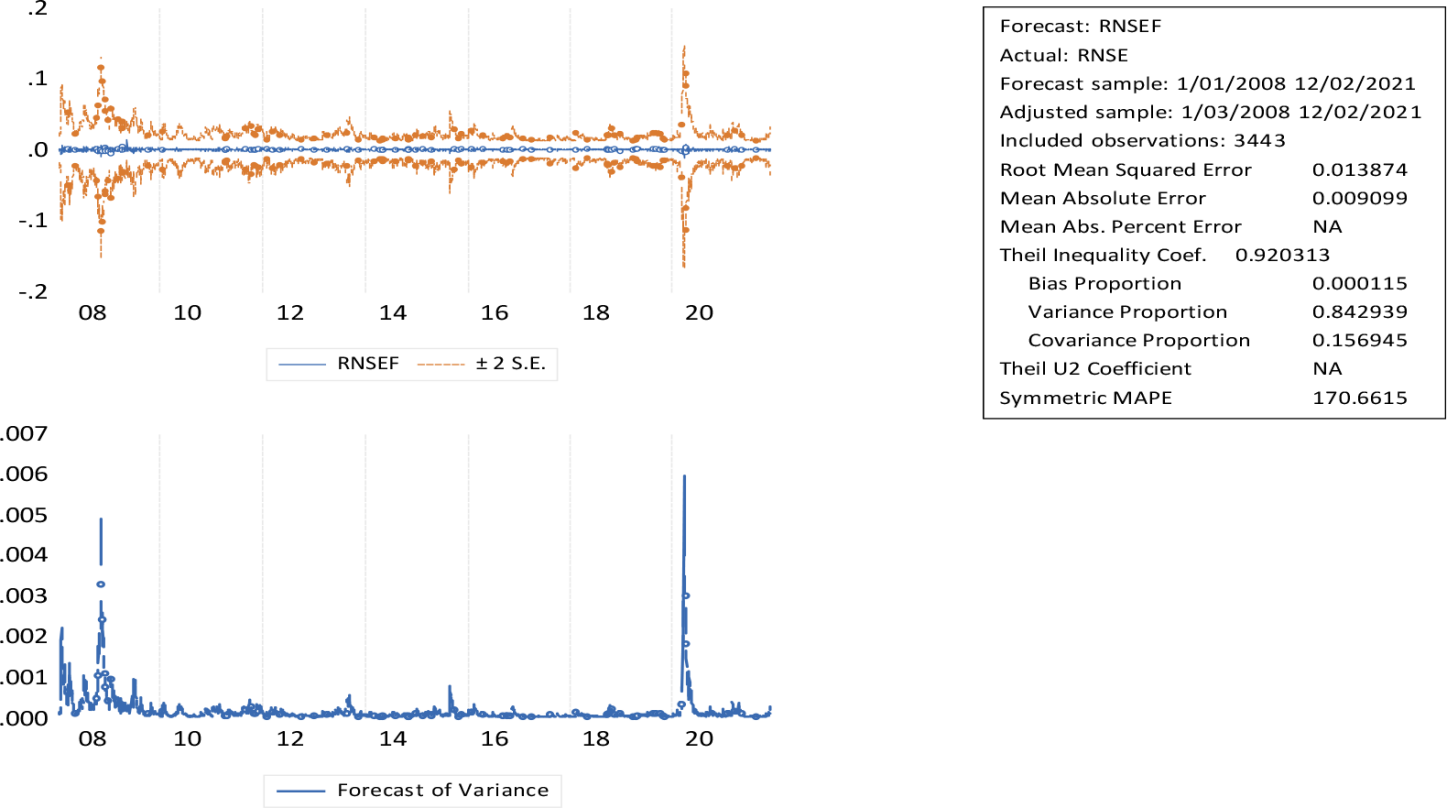
GARCH (1,1) NSE volatility forecasting and horizons (source: author’s calculations). GARCH: generalized autoregressive conditional heteroskedasticity; NSE: National Stock Exchange; RNSE: returns of National Stock Exchange.

From the above analyses it is clear that, regardless of the fit effect or estimation accuracy, GARCH models can be appropriately adapted to the volatility of the Indian stock market. Furthermore, GARCH (1,1, symmetric model), TGARCH and EGARCH (asymmetric models), perform well in our out-of-sample estimation. These empirical results can generally be categorized into two sections, beginning with the ARCH, GARCH, and FIGARCH models followed by an analysis of the TGARCH and EGARCH models using their main objectives. The preliminary analysis of the NSE indices is based on the analysis of different descriptive statistics. Table 3 demonstrates significant coefficients for constant variance, ARCH, and GARCH parameters at the 1% level. These results pertain to the GARCH heteroscedasticity model. The constant (0.00000175) was coupled with its past (0.893) and past errors (0.0.098). Our findings are consistent with Mathur *et al*. (2016). These parameters also indicate resilience of volatility shocks. Based on the estimated output, lagged volatility and fitted variance are significant. Figure 3 indicates that long-term forecast periods are associated with greater uncertainty, and short-term forecast periods are associated with lower uncertainty. Based on this study, investors can select companies in accordance with their risk aversion. The conditional volatility of the market return series from January 2008 to December 2021 shows volatility shifting across time, with violent price swings clustering around the boom. Higher prices emerged in response to solid economic fundamentals, but the real cause appears to be imperfections in the Indian market.

When comparing the findings produced using GARCH and FIGARCH, as shown in Table 4, the ARCH coefficient increases away from 1.00 whereas the GARCH coefficient falls away from 1.00. The consequences of current shocks are evident in the variance prediction for subsequent years. The difference between positive and negative news in the NSE index is 0.193, which is the asymmetric term’s coefficient. Our research revealed that the volatility of the Indian stock market could be affected asymmetrically by recent negative and positive returns, with a particularly negative rate of return resulting in greater future volatility.

## 5. Conclusion

The heteroscedasticity test confirms that there is an arch effect in the Indian stock market. Thus, the GARCH (1,1) model is justified for time-varying conditional volatility of NSE returns. As shown in Table 4, the mean equation is r_nse = 0.000691 + 0.060154. This study is based on secondary data for a period of 13 years ranging from January 2008 to December 2021.The GARCH (1,1), FIGARCH and EGARCH approaches are applied to determine the long-term persistence of volatility. Based on the results, the null hypothesis is rejected. The returns on stocks appear to be stable, though very volatile. As a result of the current shocks, future forecasts of variance are likely to be affected for several years. Information, news, and events can also significantly impact the stocks’ volatility. We observed an asymmetrical reaction in the NSE return series in response to both good and bad news. Due to the leverage effect, a negative innovation (news) would have a greater impact on volatility than a positive innovation (news). According to this stylized fact, the innovation sign significantly affects the volatility of returns and bad news increases volatility more than good news. Therefore, we conclude that bad news in the Indian stock market increases volatility more than good news. Volatility models derived from the GARCH equation provide accurate forecasts and are useful for portfolio allocation, performance measurement, and option valuation.

Moreover, it would be useful to investigate the volatility forecasting and impact of good and bad news on the inclusion of a larger sample of countries from Asia, Africa, North America and Europe in comparison to the pre- and post-crisis periods.

## Data availability

### Underlying data

Figshare: Modelling time-varying volatility using GARCH models. https://doi.org/10.6084/m9.figshare.20681203.v2 ( Ali *et al.,* 2022)

This project contains the following underlying data:

• Data.xlsx (data from the NSE-NIFTY 50 [National Stock Exchange] indices)

Data are available under the terms of the Creative Commons Attribution 4.0 International license (CC-BY 4.0).

## Footnotes

^1^
The different websites used to collect the data of Indices are www.nse.com and www.yahoofinance.com.

^2^
The generalized autoregressive conditional heteroskedasticity (GARCH) process was introduced by economist and Nobel Memorial Prize winner Robert F. Engle in 1982. The approach is intended to estimate financial markets’ volatility.
