# Supplementary material for: Modelling time-varying volatility using GARCH models: evidence from the Indian stock market
Source: F1000Res. 2022 Dec 8;11:1098. Originally published 2022 Sep 27. [Version 2] doi: 10.12688/f1000research.124998.2 (PMC9758444; doi:10.12688/f1000research.124998.2)
Supplement: Peer review history

# Peer review: Reviewer response for version 2

**DOI:** 10.5256/f1000research.141816.r157808

**Published:** 2022-12-16

**Authors:** Suleman Sarwar

**Affiliations:** 1 Finance and Economics Department, College of Business, University of Jeddah, Jeddah, Saudi Arabia

The authors have addressed all the reported comments. I have no further comments.

Is the work clearly and accurately presented and does it cite the current literature?

Partly

If applicable, is the statistical analysis and its interpretation appropriate?

Partly

Are all the source data underlying the results available to ensure full reproducibility?

Yes

Is the study design appropriate and is the work technically sound?

Yes

Are the conclusions drawn adequately supported by the results?

Yes

Are sufficient details of methods and analysis provided to allow replication by others?

Partly

Reviewer Expertise:

My area of expertise are financial economics, portfolio analysis, energy economics and environmental economics etc.

I confirm that I have read this submission and believe that I have an appropriate level of expertise to confirm that it is of an acceptable scientific standard.

# Peer review: Reviewer response for version 1

**DOI:** 10.5256/f1000research.137257.r157105

**Published:** 2022-12-02

**Authors:** Suleman Sarwar

**Affiliations:** 1 Finance and Economics Department, College of Business, University of Jeddah, Jeddah, Saudi Arabia

**Review Comments to authors:**

**Introduction:**

 1. There are a few sentences which need references, such as *“In times of crisis, investors make errors of judgment as long as a group of investors make irrational decisions, leading to the worsening of the situation in the stock market.”*

 2. In the introduction, the author has to clearly mention the main contribution of this study. See for example ^1^^,^^2^.

**Literature:**

 3. I suggest that author has to write the literature in a systematic way,

 4. I recommend some of the recent studies need to be included in literature, such as 2019, 2020 etc. ^3^^,^^4^^,^^5^^,^^6^^,^^7^.

**Data and Results:**

 If we focus on results section, it needs modifications.

 5. Why is it important to discuss this Indian Market instead of other markets?

**Conclusion:**

 6. First paragraph of conclusion is too lengthy, please report the main findings and specific (instead of general) policy implications.

Is the work clearly and accurately presented and does it cite the current literature?

Partly

If applicable, is the statistical analysis and its interpretation appropriate?

Partly

Are all the source data underlying the results available to ensure full reproducibility?

Yes

Is the study design appropriate and is the work technically sound?

Yes

Are the conclusions drawn adequately supported by the results?

Yes

Are sufficient details of methods and analysis provided to allow replication by others?

Partly

Reviewer Expertise:

My area of expertise are financial economics, portfolio analysis, energy economics and environmental economics etc.

I confirm that I have read this submission and believe that I have an appropriate level of expertise to confirm that it is of an acceptable scientific standard, however I have significant reservations, as outlined above.

## References

[1] : Quantile Granger causality between US stock market indices and precious metal prices. *Resources Policy* .2022;76: 10.1016/j.resourpol.2022.102595 10.1016/j.resourpol.2022.102595

[2] : The importance of oil assets for portfolio optimization: The analysis of firm level stocks. *Energy Economics* .2019;78: 10.1016/j.eneco.2018.11.021 217-234 10.1016/j.eneco.2018.11.021

[3] : Connectedness between energy and nonenergy commodity markets: Evidence from quantile coherency networks. *Resources Policy* .2021;74: 10.1016/j.resourpol.2021.102318 10.1016/j.resourpol.2021.102318 PMC922629435765415

[4] : Analyzing volatility spillovers between oil market and Asian stock markets. *Resources Policy* .2020;66: 10.1016/j.resourpol.2020.101608 10.1016/j.resourpol.2020.101608

[5] : Analysing volatility spillover between the oil market and the stock market in oil-importing and oil-exporting countries: Implications on portfolio management. *Resources Policy* .2019;62: 10.1016/j.resourpol.2019.03.004 22-32 10.1016/j.resourpol.2019.03.004

[6] : Volatility spillovers and hedging: Evidence from Asian oil-importing countries. *Resources Policy* .2019;61: 10.1016/j.resourpol.2018.04.010 479-488 10.1016/j.resourpol.2018.04.010

[7] : Effect of Fundamental and Stock Market Variables on Equity Return in Pakistan. *Science International* .2013;25(4) :

# Peer review: Reviewer response for version 1

**DOI:** 10.5256/f1000research.137257.r151678

**Published:** 2022-10-10

**Authors:** Diksha Panwar

**Affiliations:** 1 IILM University, Noida NCR, New Delhi, India

**The theme is intriguing, and I can see value in research:** *- “*Modelling time-varying volatility using GARCH models: evidence from the Indian stock market”- Forecasting the volatility of the Indian stock market has become essential for economic and financial analysts as a result of the financial crisis. An analysis of the NSE index returns showed that the Indian stock market had an autoregressive conditional heteroskedastic trend. The author of this paper has looked into the Indian stock market's volatility from 2008 through 2021. The GARCH equation, which is helpful for portfolio allocation, performance evaluation, and option valuation, has been utilised by them to produce precise projections.

**The introduction is well written and constructed extremely nicely: -** The introduction is so well constructed as it grabs the interest of the reader, as well as it was clear, brief, purposeful, and focused.

**The presentation of the result and discussion part has been well-organised: - **The GARCH model, which is properly fitted to the volatility of the Indian stock market, was employed by the author for this study. If we take a closer look, the study may be divided into two sections: an investigation of the TGARCH and EGARCH models based on their primary goals, and then the ARCH, GARCH, and FIGARCH models. Based on the examination of various descriptive statistics, the NSE indices' preliminary study. Lagged volatility and fitted variance are substantial based on the estimated output. If we dig deeper into the data, we find that longer prediction durations are linked to higher levels of uncertainty whereas shorter forecast periods are linked to lower levels of uncertainty. According to this study, investors can choose companies based on how risky they are. The potential instability of the market return series from January 2008 to December 2021 shows volatility shifting across time, with violent price swings clustering around the boom. 

  I certify that I have reviewed this work and possess the requisite level of knowledge to testify to its scientific quality.

*Further research can be conducted using big data analytics techniques to get more accurate forecasting of Indian stock market*

Is the work clearly and accurately presented and does it cite the current literature?

Yes

If applicable, is the statistical analysis and its interpretation appropriate?

Yes

Are all the source data underlying the results available to ensure full reproducibility?

Yes

Is the study design appropriate and is the work technically sound?

Yes

Are the conclusions drawn adequately supported by the results?

Yes

Are sufficient details of methods and analysis provided to allow replication by others?

Yes

Reviewer Expertise:

Operations, Digital Marketing, Financial Marketing. Modelling

I confirm that I have read this submission and believe that I have an appropriate level of expertise to confirm that it is of an acceptable scientific standard.
